# Notes and News

**Published:** 1887-12-31

**Authors:** 


### Notes and News.

Collected and Communicated.

We have received contributions and corrections for the "In and Out Patients' Hospital Diary" from the officials of nearly a hundred hospitals, and beg to thank these gentlemen for their courtesy.

We have received copies of the annual reports of a number of hospitals, including the Brompton Hospital for Diseases of the Chest, the Hospital for Epilepsy and Paralysis, the Ophthalmic Hospital, Moorfields, the Seamen's Hospital, the Montrose Royal Lunatic Asylum, the National Hospital for Consumption, Ventnor, etc., and hope to notice their contents shortly.

The Work Competition promises to be successful, despite the short notice. As announced elsewhere, we have decided to accept all contributions sent in to the above competition up to and including Saturday, January 7th, 1888. This course has been determined upon, owing to the omission of an announcement of the date when the competition closed in The Hospital for last week. Full particulars will be found on page 242 of the present issue, under the heading Amusements with Profit.

The Royal National Hospital for Consumption, Ventnor, has during the last year been adding to its usefulness by the erection of new houses, in memory of the late Mr. John Jones, who bequeathed to the hospital a sum which produces an income of £2,000 a year. As has been already told in our columns, this institution is conducted on the best possible principles for the relief of the disease for which it is intended, and a wise preference is given to those applicants whose cases give most promise of cure. The hospital is in part self-supporting, but depends largely on voluntary contributions, and funds are urgently required for the maintenance of the large and increasing number of patients.

Measures are being taken to ascertain the views of the local authorities in Aberdeen and district on the question of erecting for their joint use a hospital for the treatment of infectious diseases. Since the exclusion of such cases from Aberdeen Infirmary, the duty of providing accommodation for patients has been thrown on the various parishes. The difficulty in the way of small parishes coping with this statutory obligation has suggested the idea of a Combination Hospital, and the subject was informally discussed on the 16th, at a meeting in the Townhouse, Aberdeen.

The number of patients under treatment at the Seamen's Hospital, Greenwich, during 1886 was 2,095, showing an increase of nearly five hundred and fifty over the previous year. Many of these were suffering from diseases caused by exposure at sea after shipwreck—all sufferers of this kind being sent to the Seamen's Hospital by the British Consul at the first port they reach after being rescued. As usual, the subscriptions to this hospital include considerable sums collected at every port in the kingdom, and donations from foreign monarchs and Governments. The income of the hospital during last year was £11,111 1*s.* 8*d.*, and the expenditure £11,624 12*s.* 3*d.*

The committee of the London Hospital have considerately resolved to keep the post of assistant aural surgeon open as long as Mr. Mark Howell, the present holder, is in attendance on the Crown Prince of Germany.

We are requested by Sister Philippa, of King's College Hospital, to say that the letter in *The Queen* on "Rest for Nurses," from which we gave a quotation, was written by the Hon. Mrs. Armytage, and not by Mr. F. Armytage, as erroneously stated.

A new charitable institution, named the Victoria Dispensary, and specially devoted to the treatment of consumption and other diseases of the chest, has been opened in Edinburgh. The institution has been placed under the charge of Dr. R. W. Philip, whose thesis on the Etiology of Phthisis recently gained the Edinburgh University gold medal, the highest honour attainable by graduates.

The annual report of the Alexandra Hospital for Children suffering from Hip Disease, Queen-square, W.C., shows that the hospital possesses eighty beds; fifty-nine in London, and twenty-one at the "Helen" branch at Bournemouth. The committee especially desire to draw attention to the Samaritan Fund in connexion with the hospital, for paying the travelling expenses of children when sent to the Bournemouth branch. At present the fund is almost wholly supplied by one donor, and it is desired that the attention of the public at large should be directed to this truly Christian work. Complete instead of only comparative recovery may often depend on the patients enjoying a visit to the "Helen" branch hospital before returning to their homes. The committee are also desirous of building a new hospital, the cost of which would, it is estimated, be from £10,000 to £12,000. They have already acquired a site by the purchase of No. 1, Queen Square Place, which at present is used as an isolating building in the event of any infectious disease breaking out in the hospital.

The *Norwich Argus* says: "We are in a position to contradict the statement by a contemporary that the honorary medical staff of the Norfolk and Norwich Hospital contemplate sending in their resignations. What they really propose doing is as follows: Sir Peter Eade, the senior physician, and Mr. Cadge and Mr. Crosse, the two senior surgeons of the institution, have for several years filled those positions with distinction and success. In the operating-room and in the sick ward of the hospital they have combined, as anyone at all acquainted with the institution knows, experience with skill in the treatment of the scores of difficult cases which have from time to time been entrusted to their care. They are, indeed, veterans in the medical world of Norwich, and they think the time has come when they should withdraw from the more active work of the hospital, and, so to speak—and this is a consideration which carries most weight with them, and is principally responsible for the step they are about to take—make way for younger men, who will thus be afforded an extended practice, and an opportunity of rendering the hospital more real and efficient service. The Board of Management will therefore be requested to alter the existing rules of the hospital so as to allow of the partial, and, in fact, only nominal retirement, of the present senior physician and surgeons, who, in any case, will retain their valuable connexion with the institution. The proposal will not be laid before the Board until their meeting in January next, and the probability is that it will be March or April of the next year before the proposed change comes into operation. Meanwhile it is gratifying to reflect that Sir Peter and his colleagues, instead of bringing about the disaster to the hospital their full and complete retirement would doubtless have effected, are actually taking steps to secure the greater usefulness and prosperity of that noble institution."

The following vacancies are announced this week, the amount of salary in each case being given after the name of the institution :—

**Resident Medical Officers.**

City of London Hospital for Diseases of the Chest. Medical Officer.—£100.
Bootle Corporation Hospital for Infectious Diseases. Medical Officer.—£100.
Addenbrooke's Hospital, Cambridge. House Physician.—£65.
Hull Royal Infirmary. Junior Assistant House Surgeon.—£50.
Monsall Fever Hospital, Manchester. Assistant Medical Officer. £100.
Hospital for Sick Children, Ormond Street. Junior House Surgeon.—£50.

**Non-resident Medical Officers.**

Poplar Hospital for Accidents, Poplar. Two Honorary Surgeons.
St. John's Hospital for Diseases of the Skin. Two Assistant Physicians.
Essex and Colchester Infirmary. Physician.—£200.

**Matron.**

Dumfries and Galloway Royal Infirmary. Matron wanted, to commence duties immediately.—£50.

**Nurses.**

Infirmary of the Parish of Paddington. Head Nurse—£26, rising £2 annually to £30, with board, washing, and uniform.
Portsmouth Royal Hospital, Portsmouth. Head Nurse for Male Surgical Wards.—£25, with uniform.
Hospital, Weston-super-Mare. Trained Night Nurse for Male and Female Wards.—£25.
Royal National Hospital for Consumption, Ventnor. A Probationer (lady) Nurse. State age, etc.
Monsall Fever Hospital. Night Superintendent; mustbe certificated trained Nurse.—£40, with uniform.
Home for Invalids, 7, Alexandra Road, Swiss Cottage. Well-trained Nurses for outdoor work.
Nurses' Home, Norwich. Thoroughly trained Nurses for private work.

**Probationer.**

Nurses' Home, Norwich. Probationer wanted, between 25 and 35.

---

It is proposed to hold a bazaar on behalf of the building fund of Boston Cottage Hospital, to meet a deficit of £300.

---

At the anunal general meeting of the Dental Hospital of London it was stated that the number of cases treated during the year in that institution was 43,745, nearly double the number attended in 1874, when the hospital was transferred from the original premises in Soho Square to the present building in Leicester Square.

Messrs. Spiers and Pond, who were the refreshment contractors for the Liverpool Exhibition, recently closed, have presented the crockery which they had in use, numbering some 10,000 pieces, to various local hospitals and charitable institutions. The distribution of these very useful and welcome gifts was presided over by Mr. Aspinall, the Recorder of Liverpool.

---

The National Hospital for Diseases of the Heart and Paralysis, 32, Soho Square, W., is fortunate in being able to show on its balance-sheet for the year the sum of £135 6*s.* 11*d.* to its credit at the bankers after all necessary expenses have been paid. The in-patients during the year number 94, and 12,065 attendances of out-patients have been registered. There are still, however, some vacant beds in the hospital, which increased means would enable the committee to fill.

---

The North London Hospital for Consumption and Diseases of the Chest, Mount Vernon, Hampstead, which has an out-patients' branch at Tottenham Court Road, is at present heavily burdened. There is a mortgage upon the freehold of the hospital amounting to £5,000, a debt of £800 due to the bankers on account of ordinary expenses, and upwards of £1,000 due for current liabilities. The report shows a decrease of income amounting to nearly £350 compared with the preceding year; but this is due to the hospital having received a much smaller sum in legacies than in 1885. The annual subscriptions, it is satisfactory to report, show a considerable increase—£1,552 12*s.* 6*d.*, against £1,430 12*s.*

The Mayor of Dewsbury has sent a cheque for £10 to the secretary of the Infirmary, for the purpose of giving a party to the staff of that institution; the surplus, if any, to be given to the convalescent fund. It is a happy thought on the part of this gentleman to provide some share in Christmas merriment for hospital workers, who are often neglected entirely in favour of the sufferers in their charge, though no class of the community deserves or requires a little pleasure more than they.

---

At the annual meeting of the Stirling Royal Infirmary, Bailie Low entered a protest against the indiscriminate use of letters of recommendation for out-patients. He said that many mechanics and others, who could well afford to pay for medical attendance, received these recommends; thus taking the place of those who needed gratuitous care, as well as injuring the medical men of the town. If this is the case, some new regulation on the subject of out-patients should certainly be made.

---

The report of the Committee of the Hospital for Diseases of the Skin, Stamford Street, Blackfriars, for last year shows an income of £1,258 12*s.* 9*d.* and an expenditure of £1,187 7*s.* 3*d.* As, however, the institution has been burdened for some years with a debt of nearly £900, increased subscriptions are required to enable it to carry out its useful work. As it is managed on the most economical principles, and is, in a great measure, self-supporting, this hospital deserves the consideration of the charitable public.

---

A movement is on foot in Liverpool to prepare pauper children for emigration to Canada and other colonies by taking them away from the workhouse, and planting them out in preparatory homes for some time before sending them abroad. Several of these homes are already in existence in Liverpool, and are cordially supported by public men; but more are needed, and might very properly be built by the guardians themselves. It is hardly possible to say too much in praise of this scheme, which sends children abroad already endowed with some degree of that independence and self-respect which workhouse training does so little to foster, and which are so important in enabling them to become useful and successful colonists.

---

"The local branch of the Boilermakers and Iron Shipbuilders' Society," says the *Liverpool Courier*, "have adopted a plan for celebrating the Queen's Jubilee which does credit to their thoughtfulness and sense of gratitude. The scheme took the form of a jubilee voluntary gift from the members of the society to the Birkenhead and Liverpool Eye and Ear Hospitals. It is a well-known fact that members of this society are more liable to accidents, and especially to injuries of the visual and aural organs, than almost any other class of mechanics, and for the sake of the members afflicted, as well as for the sake of the society's funds, the Executive Council have found it of great advantage to send the members from all parts to Liverpool, all eye cases being treated by Dr. Browne, principal of the Eye and Ear Infirmary, and other accidents by Dr. Thomas. But for the badness of trade, there is no doubt that the amount collected for the purpose would have been much greater; as it was, the sum reached the creditable total of £21, of which £15 15*s.* was given to the Liverpool Infirmary, and £5 5*s.* to the Birkenhead Hospital. In addition to this, the Liverpool institution has been presented with a splendid electric battery, supplied by an eminent London firm, at a cost of £10 10*s.*, which will doubtless be a valuable acquisition to the medical appliances of the place. This refreshing example of the appreciation of charitable institutions on the part of workingmen deserves to be widely known and more generally copied."

The number of patients admitted into the Chelsea Hospital for Women during the past year is 398; while the number of out-patients attended is 3,450. It is to be regretted that in addition to a mortgage of £5,000, the banking account of the institution is overdrawn to the amount of £474.

The Hornsey Local Board have not yet built their hospital for infectious diseases, but they have obtained the promise of the Local Government Board to hold a local inquiry, which is a step in the right direction. Of course there is still a certain amount of opposition, but if the Local Government Board place no difficulty in the way the object will no doubt be achieved.

The Committee of the West London Hospital, Hammersmith, state in their annual report that the debt on the hospital of £7,023 has been reduced to £5,794; and that they have secured, at a cost of £2,300, the copyhold of the property which they formerly held only on lease. During the year the old drainage system of the hospital has been replaced by one in accordance with the latest improvements, and the interior of the hospital has been cleaned and painted throughout.

It is proposed to begin a Hospital Sunday Fund at Keighley, and a meeting for the purpose of discussing the question was held at the Acorn Coffee Tavern on the 6th inst. The Rector (the Rev. H. J. Longsdon), the Rev. E. Pringle, and several other gentlemen were present, and unanimously agreed to follow the example set by so many other towns, and it was decided to hold the first collection on the last Sunday in January.

Mr. J. B. Freeman, the Chairman of the Chester Working Men's Hospital Saturday Committee, sends a stirring appeal to his fellow working men, through the columns of the newspapers, not to "hold aloof and do nothing for that noble institution—the infirmary." "Start the New Year," he says, "by the weekly contribution of one penny, and don't let the names of your various firms be conspicuous by their absence when the subscription list appears." It is to be hoped that this manly appeal from one of themselves will rouse those whom Mr. Freeman addresses, to join in doing all they can for the hospitals.

After Mr. Murphy's lecture was finished, Mr. Whiteley, of Nottingham, gave some particulars of the working of the Act for compulsory notification of infectious diseases, as carried out in the town he lived in, and gave it credit for the great decrease in severity of scarlet fever epidemics. Last year the deaths from that disease numbered only 13, whereas a few years ago they amounted to 300. Some of this improvement may be due to improved sanitary arrangements and better medical attendance and nursing; but no doubt much of it is owing to the immediate and complete isolation which an Act of this nature makes possible.

At the Parkes Museum of Hygiene, on the 18th inst., Mr. Shirley Murphy gave an interesting lecture on scarlet fever. He reviewed its past history, noting how it had been confused with measles, military fever, and diphtheria. It was not till 1859 that the Registrar-General distinguished in his returns scarlet fever from diphtheria. Interesting statistics were given of the periods of the year when certain diseases were most prevalent. Measles was most prevalent at the beginning of the year, scarlet fever from September to November, and diphtheria from October to December. The lecturer also pointed out that while diphtheria had no fixed time for reappearing in an epidemic form, scarlet fever came pretty regularly every five years. Finally Mr. Murphy explained the necessary precautions to be enforced on those attending scarlet fever patients, and on families any of whose members were suffering from that disease.

There is a tendency among unthinking and penny-wise folks to grumble at the cost of hospitals for infectious diseases, as being expensive to keep up, and quite useless when infectious diseases are absent. On this point the *Sheffield Independent* says: "For our own part, we would much rather bear the burden of having it (the hospital) empty than filled; for its emptiness will be a tribute to the sanitary state of the town. Discontent at an infectious hospital out of work as being a superfluous luxury, is as ungracious as complaints by one who has insured his life, at living so long that his premiums have become a poor investment."

The Royal Ophthalmic Hospital, Moorfields, is fortunate in being one of the few London hospitals whose income more than equals its expenditure. The committee of management exercise a rigid economy in various items of outlay, but are careful not to curtail the supply of those necessaries which are required for the patients. The total number of in-patients during last year was 2,049, while the attendances of the 26,211 out-patients numbered 123,555. These figures show how extensive is the work undertaken by the Ophthalmic Hospital, a work which is increasing year by year. The total cost of maintaining the institution during the year was £6,155 11*s.* 6*d.*

The Grosvenor Hospital for Women and Children, Vincent Square, Westminster, is to a great extent a self-supporting institution, the contributions of patients having during the last year formed at least a third of its income. Patients pay from 5*s.* to £1 1*s.* a week, according to the accommodation they obtain, and to whether or not they are recommended by subscribers. It is to be regretted, however, that in spite of this the income stands at £1,002 10*s.* 5*d.*, against an expenditure of £1,083 17*s.* 3*d.*, showing a deficit of more than £80. This is the more to be regretted because the premises now occupied are, in many respects, unsuitable for a hospital, and considerable alteration and extension are desirable.

A hospital for infectious diseases has been opened at Marland, near Rochdale. The building was originally the Marland Workhouse, and was acquired by the Health Committee at the moderate price of £1,250. As the buildings cost £12,000 twenty years ago, and are still substantial, and the property includes besides three acres of freehold land, it must be admitted that they made a good bargain, especially as the alterations necessary to fit the workhouse for its new function have been so economically carried out as to leave £400 in the treasurer's hands for future repairs.

It is to be regretted that every misfortune that can befal human nature should be used up by some people as material for copy. A gentleman of that class having been attacked with scarlet fever took occasion in the columns of the *Globe* to revile the hospital where he was nursed back to health. After him, however, comes a correspondent of the same paper, who having suffered from the same disease, was taken to the London Fever Hospital, Liverpool Road, which he did not find such a dreary prison as the previous account would have made him expect. The nurses were "bright, good-tempered, and indefatigable"; "the resident doctor used to divide his spare time among the patients, and very seldom an evening passed," says the writer, "in which he did not come to my room for a game of cards, besides looking in occasionally in the afternoon." This patient enjoyed the use of the hospital library, which he found good and varied; but complains that he missed one treat. "I had the fever in the early spring. Had I postponed my illness till summer I could, as soon as I was strong enough, have played tennis every day in the large garden adjoining the hospital with the doctors and other patients." The public can judge for itse[illegible] whether the average mortal is most likely to praise or blame without reason, and so decide which of these two writers is the more worthy of belief.

